# Condom Use at Last Sex and Sexual Negotiation Among Young African American Women in North Carolina: Context or Personal Agency

**DOI:** 10.1007/s40615-023-01693-4

**Published:** 2023-07-26

**Authors:** Chukwunomso E. Osakwe, Isa van der Drift, Claudia A. Opper, William A. Zule, Felicia A. Browne, Wendee M. Wechsberg

**Affiliations:** 1 Duke University Global Health Institute, Durham, NC, USA; 2 Substance Use, Gender, and Applied Research Program, RTI International, 3040 East Cornwallis Road, PO Box 12194, NC 27709-2194 Durham, USA; 3 Gillings School of Global Public Health, University of North Carolina, Chapel Hill, NC, USA; 4 Department of Psychology, North Carolina State University, Raleigh, NC, USA; 5 Department of Psychiatry and Behavioral Sciences, Duke University School of Medicine, Durham, NC, USA

**Keywords:** Substance use, Sexual communication, Safer sex negotiation, Social and contextual factors, HIV risk-reduction, Young African American women

## Abstract

African American women are 10.8 times more likely to be diagnosed with HIV compared with White women. This descriptive study fills a gap by examining associations among social and contextual factors and sexual communication, condom use, and safer sex negotiation among African American women. Study participants between 18 and 25 years of age and who reported recent substance use were recruited from three North Carolina counties. A risk behavior survey was administered via audio computer-assisted self-interview, and logistic regression analyses were conducted to assess associations between social and contextual variables and condom use at last sex with a main partner. Education (AOR: 2.078; 95% CI: 1.214, 3.556), sexual communication with a main partner (AOR: 1.079; 95% CI: 1.050, 1.109), and condom use relationship scale (AOR: 1.059; 95% CI: 1.023, 1.098) were positively associated with condom use at last sex, whereas living with a main partner (AOR: 0.447; 95% CI: 0.210, 0.950) and the alcohol and drug problem scale (AOR: 0.971; 95% CI: 0.944, 0.998) were negatively associated with condom use (*p* < 0.05). The study findings show that among young African American women at risk for HIV, contextual and personal factors may influence condom use. A socio-ecological approach combining personal empowerment, interpersonal, structural, and biobehavioral strategies is necessary in implementing holistic gender-focused HIV prevention programs.

## Introduction

Young women’s agency with sexual protection has been the center of HIV prevention for several decades [1-3]. However, the disproportionate disparities among women of color have not changed over that period [4-6]. Despite progress in increasing condom use with casual partners, consistent condom use with main partners remains low [7]. Additionally, condom use with a main partner is important for younger people who have not settled into a long-term relationship and it becomes more salient among populations with higher prevalence of HIV. This study focuses on contextual and personal factors that influence condom use among young African American women, who are 10.8 times more likely to be diagnosed with HIV compared with White women of the same age group [8]. This elevated risk for HIV and other sexually transmitted infections (STIs) continues to be higher for African American women even after considering their individual behaviors, such as the number of sexual partners and drug use [9-11].

According to the socio-ecological model [12], individual-level risk behaviors (such as condomless sex) transmit HIV, structural factors (such as poverty) can affect the likelihood of engaging in risky behavior, and community-level factors (such as proximity to scene) can affect the probability of HIV exposure associated with risky behavior [13]. For instance, in North Carolina, African Americans have one of the highest poverty rates, with 21.5% of the African American population living below the poverty level compared with 10.5% of the White population living below the poverty level [14]. The local rates of HIV are highest among populations with the highest poverty rates. Structural- and community-level forces—such as unemployment, income inequality, and de facto segregation—that are exacerbated by racism have the potential to limit African American women’s housing options to areas where there is less access to quality healthcare services, a higher prevalence of HIV within social networks, and an increased rate of neighborhood crime [15]. Accordingly, risk of contracting HIV increases without any changes in personal sexual risk behaviors [16-18].

Violence may impact young women’s sexual risk at the interpersonal level. Gender-based violence (GBV) has been linked to increases in a woman’s likelihood of drug use, sexual risk behaviors, and HIV/STI risk [19]. Intimate partner violence directed at women, as well as neighborhood violence, can diminish a woman’s sense of power in a sexual relationship [20, 21]. Also, women who drop out of school are more likely to have romantic relationships with abusive partners [22]. At the individual level, education has been shown to have an inverse association with certain STI risk factors among young adult women [23, 24]. Recently, data collected from this same study sample of young African American women reinforced that higher educational attainment was protective for condomless sex [25]. Individual and interpersonal factors such as education, income earning power, and GBV exposure may affect a young woman’s sense of power in a relationship because a perceived or actual unequal power dynamic is correlated with women being less likely to negotiate sex practices that lower the risk of HIV transmission [26, 27].

HIV risk is influenced by an interplay between personal and contextual factors. For example, individual factors such as substance use may inhibit decision-making or hinder one’s ability to discuss or negotiate condom use. Additionally, alcohol use is associated with risky sexual behavior, such as having multiple sexual partners, condomless sex, and positive STI results among young African American women [28-30]. Also, men who use substances are less likely to use a condom during sex with their nonprimary partners [31].

While individual behaviors play an important role in sexual risk, focusing solely on personal behaviors may obfuscate the reality of sexual risk. Better understanding the factors that may influence a woman’s ability to use a condom with a main partner at all levels of the socio-ecological framework [12] may help in designing and implementing interventions for this key population.

## Study Objective

The objective of this study is to examine the association between personal and contextual factors and condomless sex with a main partner among a sample of young African American women who use substances.

## Methods

This article reports baseline data from an intervention study that tested different modes of delivering a behavioral risk-reduction intervention for young African American women. The study sites were selected in collaboration with county health departments (Durham, Guilford, and Wake Counties) based on the high rates of HIV and STIs within these counties, and their relative proximity to each other [32]. A complete description of the study procedures has been presented elsewhere [32]. The analyses here are restricted to data collected at study enrollment.

### Eligibility Criteria

Eligibility criteria for this study included (1) aged 18 to 25 years; (2) self-identify as Black and/or African American; (3) self-identify as female; (4) have had penetrative sex with a male partner without using a condom within the past 3 months; (5) have used alcohol or other drugs (AODs) in a greater quantity or for a longer period than they originally intended within the past 30 days; (6) have not tested for HIV within the past 3 months; (7) currently reside in Durham, Wake, or Guilford County, North Carolina, for at least the past 6 months; (8) have no intent to move from the area within the next year; (9) have not participated in the previous studies or the formative phase of this study; (10) being willing to test for HIV, chlamydia, and gonorrhea through their respective county health departments and sign a release; and (11) being willing to provide locator information for future contact.

### Recruitment

The study used a combination of recruitment strategies, including street-based outreach and marketing efforts that have been successful in our previous studies [33-35]. Project staff visited “hotspots” such as local colleges and universities, bus stops, and shopping centers and distributed recruitment flyers and cards. Recruitment also occurred in each health department. These in-person approaches were supplemented with recruitment through social media platforms and local radio advertisements. Additionally, a peer consultant program allowed for study participant referrals from participants and nonparticipants. Participants received a $50 gift card following their interview as compensation for their time. Childcare was provided for women with children.

### Measures

All participants completed an adapted Revised Risk Behavior Assessment [36] via an audio computer-assisted self-interview (ACASI). The questionnaire included sections on demographics, mental health, AOD use, substance use treatment, neighborhood disorder, sexual activity, conflict and victimization, criminal justice involvement, condom use communications and sexual discussion with main partner, power and empowerment, HIV testing, physical health and nutrition, and need for health and social services. Interviews lasted an average of 45 minutes (standard deviation 20 minutes).

### Dependent Variable

The dependent variable was derived from two items: The last time you had sex with your main partner did you use a male condom? and The last time you had sex with your main partner did you use a female condom? If a participant responded yes to either question, the response was coded as using a condom at last sex with main partner. Condom use at last sex with main partner is a common valid proxy for predicting condom use behaviors for longer time periods [37]. In this article we chose condom use at last sex with main partner because we were specifically looking at the influence of negotiation with main partner on condom use, and previous research has found that many people use condoms with casual partners but not with main partners [38] [39]. Focusing on last sex has been shown to increase validity and to reduce recall bias [37].

### Independent Variables

Neighborhood disorder was measured with a 12-item scale. Possible scores for each item ranged from 0 (never) to 3 (often), and possible scores for the scale ranged from 0 to 36. Items included questions regarding the frequency of fights that involved a gun or knife, violent arguments between neighbors, drinking in public, people selling drugs, stolen cars, gang fights, sexual assaults or rapes, robberies, burglaries, drive-by shootings, murders, and arson.

AOD use problems were measured with a 15-item scale. Items assessed the recency of different AOD-related problems. Responses ranged from 1 (in the past 30 days) to 3 (1 or more years ago). For these analyses, the coding was reversed, such that higher scores are associated with more recent problems. Possible scores on the scale ranged from 15 to 45. Examples of items in the scale include the following: You used larger amounts of alcohol or drugs or used them for a longer time than you planned? You tried to use less alcohol or drugs (cut back) but could not? You spent a lot of time using alcohol or drugs? You spent a lot of time recovering (trying to feel better) after using alcohol or drugs?

Sexual relationship with their current main partner was measured with an 8-item scale. Item responses ranged from 1 (strongly agree) to 5 (strongly disagree). Possible scores for the scale ranged from 8 to 40.

Sexual communication with their current main partner was measured with a 14-item scale. Item responses ranged from 1 (not at all) to 4 (all the time), with possible scores for the scale ranging from 14 to 56. Items inquired about the frequency in the past 6 months of communications and behaviors with their partner about specific topics. Examples of items include the following: In the past 6 months how often did you… Ask your main partner to use a male condom? Ask your main partner about using a female condom? Use a condom even if you were high/drunk? Use a condom even if your boyfriend was high/drunk? Ask if he has had sex with someone else?

### Analysis

Descriptive statistics were calculated to describe the sample. Logistic regression analyses were used to assess associations between each of the independent variables and condom use at last sex with their main partner. Variables with *p*-values < 0.10 were entered into a multiple logistic regression analysis, in addition to the county of enrollment variable. Variables with *p*-values of < 0.05 were considered statistically significant. Analyses were performed with SPSS v25.

## Results

A total of 652 participants were enrolled in the study between January 2017 and July 2019. These analyses are restricted to the 405 participants (62%) who reported having a main partner at baseline. [32]

### Characteristics of the Sample

Each of the three counties—Durham, Guilford, and Wake—contributed approximately one-third of the sample. The mean age of participants was 21.3 years and 51% had completed some higher education beyond high school. Among participants, 16% reported being currently homeless, 20% reported living with a main partner, and 35% reported full-time employment. Overall, 24% of participants reported using a condom the last time they had sex with their main partner. Over Forty-three percent of participants reported being intoxicated just before or during the last time that they had sex with their main partner, and 42% reported that their main partner was intoxicated when they last had sex. Table 1 presents more detailed characteristics of the study participants.

### Bivariate Associations Between Independent Variables and Condom Use at Last Sex with Main Partner

In bivariate analyses using logistic regression, higher education, higher scores on the condom use relationship scale, and higher scores on the sexual communication scale were all significantly (*p* < 0.05) associated with using a condom at last sex with a main partner. Living with a main partner was significantly (*p* < 0.05) associated with not using a condom (Table 2).

### Results of Multivariable Analyses

Adjusting for county in multivariable analyses, those with a greater level of education (adjusted odds ratio [AOR] = 2.078; 95% confidence interval [CI] = 1.214, 3.556), higher scores on the condom use and relationship scale (AOR = 1.059; 95%; CI = 1.023, 1.098), and the sexual communication with main partner scale (AOR = 1.079; 95% CI = 1.050, 1.109) were all independently associated with increased odds of using a condom at last sex with a main partner. Living with a main partner (AOR = 0.447; 95% CI = 0.210, 0.950) and higher scores on the alcohol and drug problem scale (AOR = 0.971; 95% CI = 0.944, 0.998) were significantly associated with decreased odds of using a condom at last sex.

## Discussion

This study highlights the complexities of HIV risk and condom use within main partnerships for young African American women. Condoms can prevent HIV/STI transmission when they are used consistently and correctly, and condom use is associated with women’s ability to negotiate for safer sex with their partners [40]. African American women who demonstrate a sense of self-efficacy, possess negotiating power, and engage in effective sexual communication with their partners are more likely to use a condom [41-45]. Many HIV studies have examined relational and structural factors [46] that influence condom use among African American women who use substances, engage in sexually concurrent relationships, and have abusive partners [35, 47-50]. However, relatively few studies have examined condom use among young African American women who are not classified as one of these key population groups [51], such as women who conduct sex work or women who inject drugs. The study findings show that among young African American women at risk for HIV, contextual and personal factors may influence condom use and help fill this gap in the literature.

In the present study, education, sexual communication with a main partner, and the condom use relationship scale were positively associated with condom use at last sex. This aligns with other studies showing that education is a protective factor for substance use [52, 53] and sexual risk [54] among adolescents and young adults [25]. Higher educational level is associated with increased ability to engage in sexual communication with a main partner [55]. The ability to engage in sexual communication with a main partner about condom use also increases the likelihood of using a condom [56, 57]. Contextual factors and personal agency can influence a woman’s ability to reduce HIV risk behaviors, as seen in studies showing that young women who lacked food, [58, 59] were homeless [60], and those with low educational level were more likely to engage in sexual risk behaviors [61-64]. Agency is key to negotiating condom use because a perceived sense of equality in a relationship enhances the ability to engage in sexual communication with one’s main partner. Consequently, women-centered interventions based on empowerment and feminist theory, such as the gender-focused Young Women’s CoOp [32], are needed for this subpopulation to reduce HIV risk through skill-building activities that enhance communication and negotiation skills [65].

Living with a main partner was negatively associated with condom use, which aligns with previous studies showing that African American women are less likely to use condoms with their main partner because of a perceived lower risk for HIV/STIs when having sex with people they cohabitate with and/or monogamous partners, as compared with casual sexual partners [60, 66, 67]. In some cases, women may be less likely to use condoms with main partners because of fear of violent reactions or retaliation in the form of resource deprivation [68, 69]. In these instances, previous studies have indicated that perceived unequal power dynamics and financial dependence [70, 71] may impede condom-use negotiations [69, 72, 73]. This is concerning because African American women are also more likely to living in communities where there are higher numbers of persons with HIV, and they are more likely to have sexual contact with individuals who have multiple sex partners [17, 26].

African Americans also have the highest gender-ratio imbalance in the USA [74]. A shortage of male partners and men’s negotiating power in a relationship are some of the factors that influence sexual decision-making and HIV risk behavior among college-educated African American women [66]. This increases their risk of contracting HIV even when they do not display sexual risk behaviors beyond condomless sex. Empowerment interventions that promote economic independence, enhance communication/negotiation skills, challenge gender stereotypes, and promote gender-equality policy solutions can help combat the unequal lines of power (race and gender) that may risk women’s health and autonomy.

AOD use was associated with not using a condom at last sex. This aligns with previous studies showing that substance use may lead to disempowerment and limit the ability to negotiate for condom use [75]. Being under the influence of AODs could lower sexual communication with a main partner and limit agency to negotiate for safer sex. However, the present study is unique because participants were not required to have alcohol use disorder (AUD) or substance use disorder (SUD) to be eligible to participate. Nonetheless, 43% of participants reported being intoxicated the last time that they had sex with their main partner, and 42% reported that their main partner was intoxicated when they last had sex. This is a significant finding and should not be overlooked because sex while intoxicated was quite common among those with any risk. It shows that even among sub-populations that may not have high rates of AUD or SUD, there is a need to consider episodic substance use and how it may influence sexual risk. These populations may benefit from increased awareness on the risks associated with being intoxicated when having sex.

Scientific advancements have led to the development of pre-exposure prophylaxis (PrEP) [76] and long-acting reversible contraceptives (LARC) [77], which are extremely helpful in preventing HIV and unintended pregnancies, respectively. However, neither PrEP nor LARC protects against other STIs. Also, PrEP is not easily accessible to some populations who need it [78, 79]. In our study population, some African American women tested positive for STIs. This suggests that it will be important to continue to encourage people to use condoms even as PrEP use increases. It will be especially important to promote awareness about and access to female condoms, which allow women the freedom to exercise personal choice in engaging in safer sex. Female condoms also cover more of the vulva, which helps prevent STI transmission [80, 81].

To make significant progress in reducing HIV risk among African American women, future interventions should strive to incorporate biobehavioral approaches combined with structural components to address the complexities of HIV and STI risk within this population. In designing interventions, we must consider all levels of influence on the socio-ecological framework [82]. Community level factors such as relationship power and gender roles should be analyzed to further understand how they influence condom negotiation ability. There is also a need for more evaluations of interventions that incorporate violence prevention to identify and document lessons learned for future applications [19]. Although neighborhood disorder was not significantly associated with condom use in this study, previous studies have found that environmental factors can influence HIV risk [83, 84]. Conducting a contextual gender analysis prior to an intervention can help to better understand gender power dynamics within a population and the contextual and personal factors that influence sexual risk behavior.

## Study Limitations

It is important to note that the study findings are limited by the reliance on self-reported data, which is susceptible to a social desirability bias. However, data were collected using ACASI technology, which has been shown to increase the validity of self-reports of potentially embarrassing or stigmatizing behaviors [85]. Additionally, the dependent variable, condom use at last sex, was selected to reduce recall bias and increase reliability. Also, although participants were recruited using a variety of methods, the representativeness of the sample is unknown, and caution should be used in generalizing the findings to other populations. Nonetheless, this represents a relatively large sample of an understudied population that was recruited from three counties. As with most intervention studies, the study design focused on increasing internal validity rather than external validity. One potential source of bias is that women who enrolled in the study also had to visit a health department clinic.

## Conclusion

The findings from this study reinforce findings from previous research underscoring the critical role of personal agency and contextual factors in reducing HIV risk among African American women. This emphasizes the importance of addressing individual and relational factors rather than simply categorizing them as a high-risk group based on risk behaviors.

To achieve the national HIV strategy of reducing HIV transmissions in the USA by 75% in 2025 and by 90% in 2030 [86], it is crucial to identify and address the key populations that have been overlooked or excluded from interventions and research. Understanding the unique circumstances by young African American women is paramount for designing more effective interventions. They constitute a key population, often due to environmental and social factors. Therefore, interventions aimed at reducing HIV risk must adopt a holistic approach, addressing factors at all levels of the socio-ecological framework and incorporating activities that empower individuals while employing gender-focused approaches. As science advances toward biobehavioral and structural approaches [87] to address HIV risk, this study reaffirms the imperative and significance of such an approach. By acknowledging the complex interplay of personal and contextual factors, interventions can be tailored to suit the specific needs of young African American women, contributing to the overall goal of ending the HIV epidemic.

To accomplish the objectives outlined in the national strategy, it is essential that research and interventions move beyond a one-size-fits-all approach and pay careful attention to the intersecting factors that shape HIV risk among different populations. By doing so, we can promote targeted and impactful interventions that address the unique challenges faced by young African American women and work toward achieving the ambitious goals set forth in the national HIV strategy.

## Figures and Tables

**Table 1:** Characteristics of the study participants

	Total *N* (%) 405 (100%)
County	
Durham	139 (34.3)
Guilford	129 (32.9)
Wake	137 (33.8)
Sociodemographic characteristics	
Mean age in years (SD)	21.3 (2.2)
Greater than a high school education	207 (51.1)
Currently homeless	65 (16.0)
Living with main partner	82 (20.2)
Sources of support	
Employed full time or part time	240 (59.3)
Employed full time	140 (34.6)
Supports self	283 (69.9)
Support from main partner	164 (40.5)
Support from own parent or guardian	171 (42.2)
Support from siblings	42 (10.4)
Support from other family members	63 (15.6)
Support from friends	43 (10.6)
Support from government (e.g., WIC, welfare)	42 (10.4)
Used condom at last sex with main partner	96 (23.8)
Intoxicated at last sex	
Participant intoxicated at last sex with main partner	175 (43.2)
Main partner intoxicated at last sex at baseline	171 (42.2)
Mean neighborhood disorder scale score (SD)	6.1 (7.9)
Mean alcohol and drug problem scale score (SD)	23.6 (9.9)
Mean condom use and relationship with main partner scale score (SD)	13.0 (9.1)
Mean sexual communication score with main partner scale score (SD)	23.3 (7.9)

*SD*, standard deviation

*WIC*, Special Supplemental Nutrition Program for Women, Infants, and Children

**Table 2:** Unadjusted and adjusted associations between independent variables and condom use at last sex with main partner

	Unadjusted OR (95% CI)	Adjusted AOR (95% CI)
County		
Durham	Ref	Ref
Guilford	2.346 (1.289, 4.217)	1.900 (0.970, 3.720)
Wake	2.079 (1.143, 3.781)	1.845 (0.952, 3.572)
Sociodemographic characteristics		
Age	0.900 (0.809, 1.000)^†^	0.913 (0.803, 1.039)
Greater than a high school education	1.837 (1.148, 2.9238)*	2.078 (1.214, 3.556)**
Currently homeless	0.774 (0.402, 1.492)	
Living with main partner	0.383 (0.189, 0.775)**	0.447 (0.210, 0.950)*
Sources of support	
Employed full time or part time	0.850 (0.535, 1.351)	
Employed full time	0.679 (0.411, 1.121)	
Supports self	0.726 (0.447, 1.180)	
Support from main partner		
Support from own parent or guardian	1.431 (0.903, 2.268)	
Support from siblings	0.506 (0.206, 1.239)	
Support from other family members	1.115 (0.600, 2.073)	
Support from friends	1.120 (0.542, 2.317)	
Support from government (e.g., WIC, welfare)	0.404 (0.154, 1.059) ^†^	0.688 (0.243, 1.943)
Intoxicated at last sex	
Participant intoxicated at last sex with main partner	1.096 (0.690, 1.742)	
Main partner intoxicated at last sex	1.156 (0.728, 1.838)	
Neighborhood disorder scale score	0.994 (0.965, 1.024)	
Alcohol and drug problem scale score	0.978 (0.954, 1.003) ^†^	0.971 (0.944, 0.998)*
Condom use and relationship with main partner scale	1.031 (1.000, 1.063)*	1.059 (1.023, 1.098)**
Sexual communication score with main partner scale	1.069 (1.043, 1.096)***	1.079 (1.050, 1.109)***

*WIC*, Special Supplemental Nutrition Program for Women, Infants, and Children

† *p*< 0.10

* *p* < 0.05

** *p* < 0.01

*** *p* < 0.001

## Data Availability

Data are available upon request.
